# Nomogram prediction for the 3-year risk of type 2 diabetes in healthy mainland China residents

**DOI:** 10.1007/s13167-019-00181-2

**Published:** 2019-08-06

**Authors:** Kun Wang, Meihua Gong, Songpu Xie, Meng Zhang, Huabo Zheng, XiaoFang Zhao, Chengyun Liu

**Affiliations:** 1 Department of Geriatrics, Union Hospital, Tongji Medical College, Huazhong University of Science and Technology, Wuhan, 430022 China; 2 Department of Clinical Laboratory, The Third People Hospital of Jimo, Jimo, 266000 Shandong China; 3 The First People’s Hospital of Jiangxia District, Wuhan City & Union Jiangnan Hospital, HUST, Wuhan, 430200 China

**Keywords:** Type 2 diabetes mellitus (T2DM), Nomogram, Risk factor, Predictive preventive personalized medicine

## Abstract

**Aims:**

To develop a precise personalized type 2 diabetes mellitus (T2DM) prediction model by cost-effective and readily available parameters in a Central China population.

**Methods:**

A 3-year cohort study was performed on 5557 nondiabetic individuals who underwent annual physical examination as the training cohort, and a subsequent validation cohort of 1870 individuals was conducted using the same procedures. Multiple logistic regression analysis was performed, and a simple nomogram was constructed via the stepwise method. Receiver operating characteristic (ROC) curve and decision curve analyses were performed by 500 bootstrap resamplings to assess the determination and clinical value of the nomogram, respectively. We also estimated the optimal cutoff values of each risk factor for T2DM prediction.

**Results:**

The 3-year cumulative incidence of T2DM was 10.71%. We developed simple nomograms that predict the risk of T2DM for females and males by using the parameters of age, BMI, fasting blood glucose (FBG), low-density lipoprotein cholesterol (LDLc), high-density lipoprotein cholesterol (HDLc), and triglycerides (TG). In the training cohort, the area under the ROC curve (AUC) showed statistical accuracy (AUC = 0.863 for female, AUC = 0.751 for male), and similar results were shown in the subsequent validation cohort (AUC = 0.847 for female, AUC = 0.755 for male). Decision curve analysis demonstrated the clinical value of this nomogram. To optimally predict the risk of T2DM, the cutoff values of age, BMI, FBG, systolic blood pressure, diastolic blood pressure, total cholesterol, LDLc, HDLc, and TG were 47.5 and 46.5 years, 22.9 and 23.7 kg/m^2^, 5.1 and 5.4 mmol/L, 118 and 123 mmHg, 71 and 85 mmHg, 5.06 and 4.94 mmol/L, 2.63 and 2.54 mmol/L, 1.53 and 1.34 mmol/L, and 1.07 and 1.65 mmol/L for females and males, respectively.

**Conclusion:**

Our nomogram can be used as a simple, plausible, affordable, and widely implementable tool to predict a personalized risk of T2DM for Central Chinese residents. The successful identification of at-risk individuals and intervention at an early stage can provide advanced strategies from a predictive, preventive, and personalized medicine perspective.

**Electronic supplementary material:**

The online version of this article (10.1007/s13167-019-00181-2) contains supplementary material, which is available to authorized users.

## Introduction

The prevalence of type 2 diabetes mellitus (T2DM) is increasing rapidly worldwide. According to the latest indication given by the International Diabetes Federation, 451 million adults suffered from diabetes worldwide in 2017, and this figure is expected to increase to 693 million by 2045 [1]. With 109.6 million patients with diabetes, China has the highest number of cases of this disease worldwide [2]. T2DM and the increased incidence of its complications are among the leading causes of death and cause huge burden to patients, especially those living in underdeveloped or developing countries [3]. What is worse, the morbidity of complications of T2DM showed a trend with much younger cases in recent years [4].

T2DM can be prevented or delayed by lifestyle and/or pharmacological interventions [5, 6]. The White Paper of the “European Association for Predictive, Preventive and Personalised Medicine (PPPM)” (EPMA) [4] suggested that a central component of preventive strategies is identification of individuals at risk for development of T2DM. The risk factors for T2DM, such as aging and obesity, can interact precisely and affect the disease by complex processes. It is known that today there are no specific methods of prediction, prevention, and treatment of T2DM, and they are therefore still to be in the focus of clinical research.

Prediction models are tools that integrate risk factors and have been identified as a practical means of identifying people for preventive interventions. A nomogram can provide accurate and individualized risk predictions for each individual. Given that a prediction model is inappropriate for utilization in other populations [7], numerous T2DM prediction models have been developed in different countries and ethnicities [8–10]. However, at present, only a limited number of related studies have been conducted in mainland China, and no study has been performed in Central China [8, 11, 12].

This study aimed to develop a precise personalized T2DM prediction model by cost-effective and readily accessible parameters in the Central Chinese population, and to provide evidence about the functional link between the selected risk factors and T2DM.

## Methods

### Study population and follow-up evaluation

In this cohort study, participants were local residents whose annual physical examinations were conducted in Wuhan Union Hospital, which is one of the largest hospitals in Central China. We collected the data of participants who visited the hospital from January 1, 2010, to May 31, 2010, as the training cohort and those from June 1, 2013, to July 31, 2013, as the verification cohort. Baseline-excluding criteria included (1) individuals with known diabetes, (2) subjects with baseline incomplete sociodemographic and clinical data, and (3) patients with history of cancer, cardiovascular disease, or stroke.

**Fig. 1:**
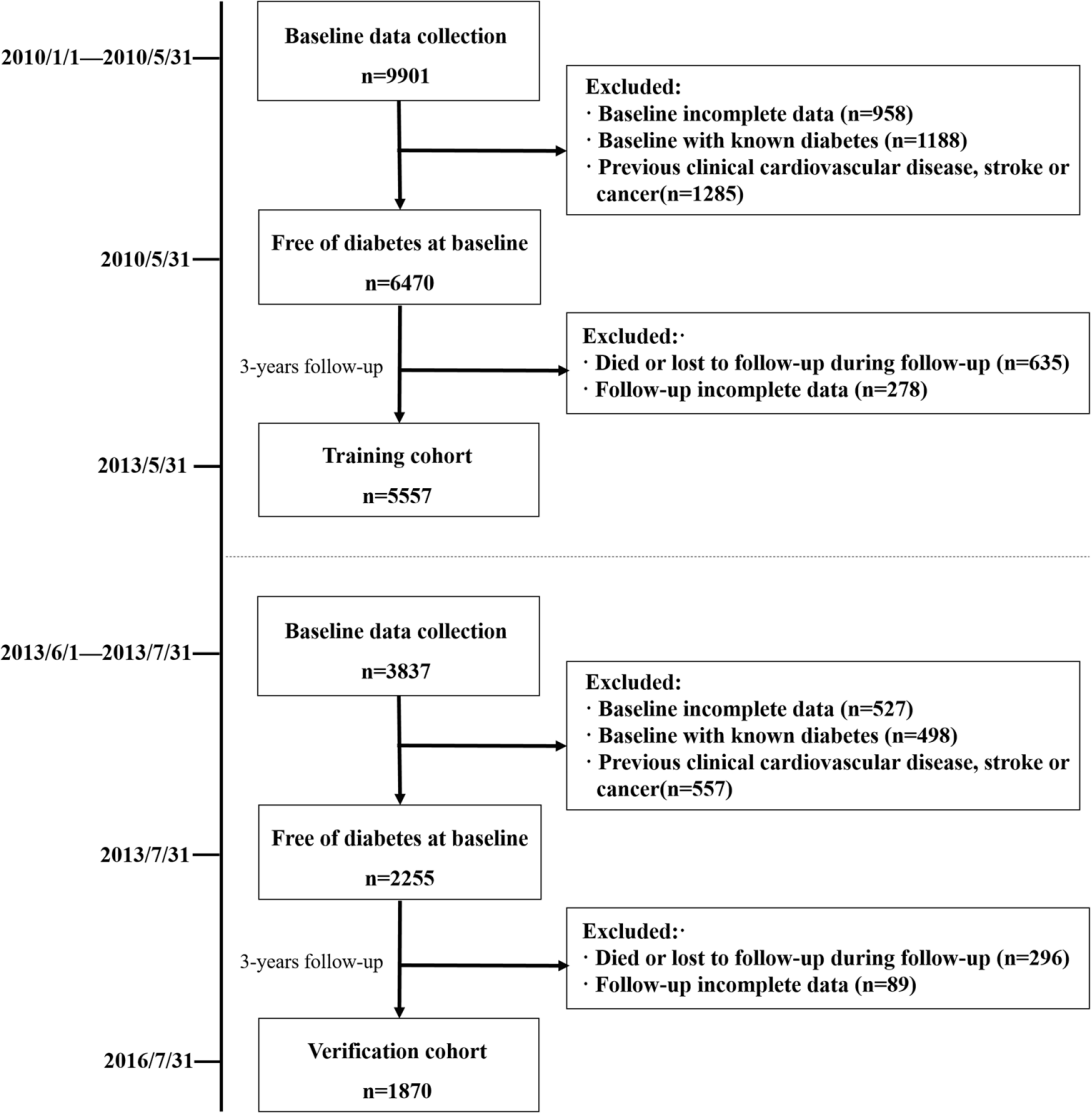
Flowchart of participants included in this 3-year cohort study

We continued collecting the physical examination data until May 31, 2013, for the training cohort and July 31, 2016, for the verification cohort. Finally, 5557 and 1870 participants were included in the final analysis as training and verification cohorts, respectively. We marked a participant as incident T2DM cases if he/she developed T2DM during the follow-up time. A flow diagram is showed in Fig. 1.

The data of these participants were used to generate prediction models for T2DM development. The physical examination data collection in Wuhan Union Hospital for this study was approved by the Tongji Medical College Ethics Committee. Verbal informed consent was obtained from each participant and was recorded by the physician who explained the study procedures. Written informed consent was not required because the data were anonymous, and the study was an observational one. [13]

### Variable measurement

Previous medical history and demographic characteristic for every subject were obtained by trained nurses through a standard questionnaire. Height was measured to the nearest 0.5 cm and weight to the nearest 0.1 kg using a height-weight scale that had been calibrated before using and with participants wearing light clothes and without shoes. The body mass index was calculated from weight (kg)/height^2^ (m^2^). Blood pressure was measured three times in the right arm in the sitting position using a mercury sphygmomanometer, with arms supported and positioned at the level of the heart after a rest period of at least 5 min.

After an overnight fast of at least 10 h, blood samples were collected in the morning and were processed within 2 h. The plasma glucose, total cholesterol (TC), low-density lipoprotein cholesterol (LDLc), high-density lipoprotein cholesterol (HDLc), and triglycerides (TG) were measured by automated chemistry analyzers (Beckman Coulter chemistry analyzer AU5800 series, Tokyo, Japan). A standard 75-g oral glucose tolerance test (OGTT) was performed next, after which a second blood sample was collected for plasma glucose measurement.

Meeting any of the following criteria can be defined as T2DM: (1) use of anti-diabetic medications, (2) fasting blood glucose (FBG) ≥ 7.0 mmol/L (126 mg/dL), or (3) 2-h postprandial plasma glucose (2hPG) ≥ 11.1 mmol/L (200 mg/dL).

### Statistical analysis

Characteristics of all participants stratified by training/validation cohort were presented as means (standard deviations) or medians (interquartile ranges) for continuous variables, and as frequencies (percentage) for categorical variables. The one-way ANOVA and Kruskal-Wallis tests were used to analyze differences between groups for normally and skewed distributed continuous variables, respectively, and the chi-squared test was conducted for analyzing categorical variables (Table 1). Baseline characteristics of the training cohort stratified by incidence of T2DM are presented in Table 2. Risk factors for T2DM were analyzed by univariate and multivariate logistic regression analysis with generalized estimating equations (Tables 2 and 3).

**Table 1:** Characteristics of the training and validation cohorts (*N* = 7427)

Characteristic	Training cohort (*n* = 5557)	Validation cohort (*n* = 1870)	*P* value
Age (years)	44.32 ± 13.42	44.33 ± 13.36	0.852
Gender, no. (%)			0.778
Female	2226 (40.06%)	756 (40.43%)	
Male	3331 (59.94%)	1114 (59.57%)	
BMI (kg/m^2^)	23.64 ± 3.19	23.65 ± 3.25	0.852
FBG **(**mmol/L**)**	4.96 ± 0.54	4.93 ± 0.52	0.104
SBP (mmHg)	117.24 ± 15.96	116.84 ± 15.95	0.356
DBP (mmHg)	77.88 ± 10.21	77.70 ± 10.27	0.497
TC (mmol/L)	4.79 ± 0.82	4.80 ± 0.83	0.531
LDLc (mmol/L)	2.49 ± 0.66	2.49 ± 0.65	0.868
HDLc (mmol/L)	1.52 ± 0.36	1.53 ± 0.35	0.467
TG (mmol/L)	1.27 (0.88–1.89)	1.27 (0.90–1.86)	0.721
Incident T2DM, no. (%)	595 (10.71%)	206 (11.02%)	0.710

Data are shown as means ± SD, median (interquartile range), or no. (%)

*BMI* body mass index, *FBG* fasting blood glucose, *SBP* systolic blood pressure, *DBP* diastolic blood pressure, *TC* total cholesterol, *LDLc* low-density lipoprotein cholesterol, *HDLc* high-density lipoprotein cholesterol, *TG* triglycerides, *T2DM* type 2 diabetes mellitus

**Table 2:** Baseline characteristics according to the incidence of T2DM and the univariate logistic regression analysis in the training cohort (*N* = 5557)

Characteristic	Incident T2DM at 3-year follow-up			Univariate logistic regression analysis	
	No (*n* = 4962)	Yes (*n* = 595)	*P* value	OR (95% CI)	*P* value
Age (years)	43.26 ± 13.11	53.14 ± 12.70	< 0.001	1.05 (1.04, 1.06)	< 0.001
Gender, no. (%)			< 0.001		< 0.001
Female	2071 (41.74%)	155 (26.05%)		1.0	
Male	2891 (58.26%)	440 (73.95%)		2.03 (1.68, 2.46)	
BMI (kg/m^2^)	23.46 ± 3.15	25.16 ± 3.04	< 0.001	1.18 (1.15, 1.21)	< 0.001
FBG (mmol/L)	4.92 ± 0.50	5.29 ± 0.69	< 0.001	3.07 (2.65, 3.55)	< 0.001
SBP (mmHg)	116.30 ± 15.56	125.03 ± 17.07	< 0.001	2.03 (1.88, 2.19)	< 0.001
DBP (mmHg)	77.48 ± 10.09	81.18 ± 10.62	< 0.001	1.03 (1.03, 1.04)	< 0.001
TC (mmol/L)	4.74 ± 0.79	5.20 ± 0.88	< 0.001	1.03 (1.03, 1.04)	< 0.001
LDLc (mmol/L)	2.46 ± 0.64	2.74 ± 0.70	< 0.001	1.94 (1.75, 2.15)	< 0.001
HDLc (mmol/L)	1.53 ± 0.36	1.46 ± 0.37	< 0.001	0.58 (0.45, 0.74)	< 0.001
TG (mmol/L)	1.21 (0.85–1.76)	1.93 (1.33–2.70)	< 0.001	1.40 (1.32, 1.48)	< 0.001

Data are shown as means ± SD, median (interquartile range), or no. (%) *P* value; odds ratio (95% CI), *P* value

*T2DM* type 2 diabetes mellitus, *BMI* body mass index, *FBG* fasting blood glucose, *SBP* systolic blood pressure, *DBP* diastolic blood pressure, *TC* total cholesterol, *LDLc* low-density lipoprotein cholesterol, *HDLc* high-density lipoprotein cholesterol, *TG* triglycerides

**Table 3:** Multivariate logistic regression analysis for risk factors associated T2DM in the training cohort (*N* = 5557)

	Female		Male	
Characteristic	OR (95% CI)	*P* value	OR (95% CI)	*P* value
Age (years)	1.07 (1.05, 1.09)	< 0.0001	1.04 (1.03, 1.05)	< 0.0001
BMI (kg/m^2^)	1.08 (1.02, 1.15)	0.0130	1.08 (1.04, 1.12)	0.0002
FBG (mmol/L)	2.38 (1.68, 3.38)	< 0.0001	1.83 (1.53, 2.20)	< 0.0001
SBP (mmHg)	1.00 (0.98, 1.02)	0.9246	1.00 (0.99, 1.01)	0.8845
DBP (mmHg)	1.00 (0.97, 1.03)	0.9534	1.00 (0.99, 1.02)	0.9778
TC (mmol/L)	1.48 (0.72, 3.04)	0.2876	1.44 (0.97, 2.15)	0.0694
LDLc (mmol/L)	0.91 (0.41, 2.01)	0.8214	1.10 (0.71, 1.71)	0.6745
HDLc (mmol/L)	1.20 (0.56, 2.57)	0.6378	0.56 (0.35, 0.91)	0.0193
TG (mmol/L)	1.50 (1.18, 1.92)	0.0011	1.16 (1.03, 1.30)	0.0110

Data are shown as odds ratio (95% CI), *P* value

*T2DM* type 2 diabetes mellitus, *BMI* body mass index, *FBG* fasting blood glucose, *SBP* systolic blood pressure, *DBP* diastolic blood pressure, *TC* total cholesterol, *LDLc* low-density lipoprotein cholesterol, *HDLc* high-density lipoprotein cholesterol, *TG* triglycerides

**Fig. 2:**
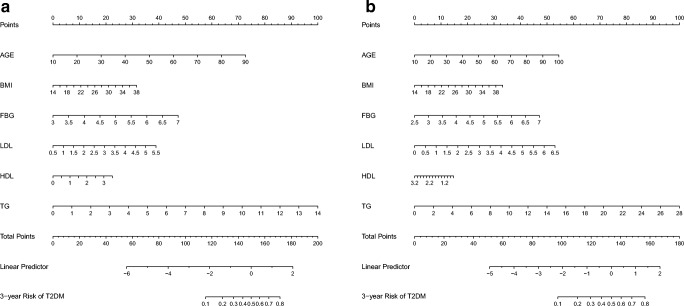
Nomogram to predict the 3-year risk of T2DM for females (**a**) and males (**b**). *Instructions: to estimate an individual’s 3-year risk of T2DM, locate his/her value on each variable axis. Draw a vertical line from that value to the top Points scale for determining how many points are assigned by that variable value. Then, the points from each variable value are summed. Locate the sum on the Total Points scale and vertically project it onto the bottom axis, thus obtaining a personalized 3-year risk of T2DM. *Using bootstrap resampling (times = 500)

**Fig. 3:**
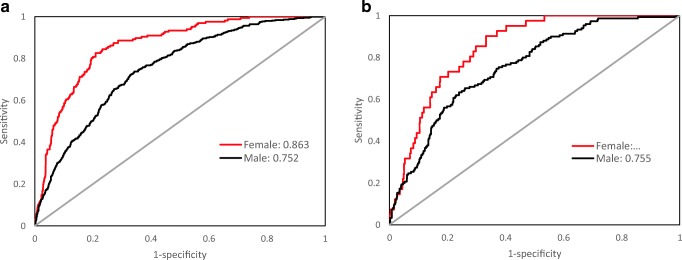
The ROC curves of the nomogram for 3-year T2DM risk in the training cohort (**a**) and validation cohort (**b**). *a In the training cohort, the AUCs of females and males were 0.863 (95% CI, 0.837–0.888) and 0.751 (95% CI, 0.729–0.774), respectively. **b** In the validation cohort, the AUCs of females and males were 0.847 (95% CI, 0.801–0.892) and 0.755 (95% CI, 0.717–0.794), respectively. ROC: receiver operating characteristics curves, AUC: area under curve. *Using bootstrap resampling (times = 500)

**Fig. 4:**
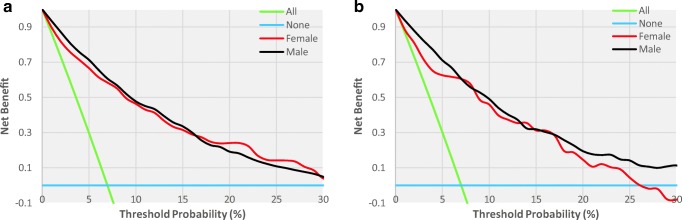
The decision curve analysis of the nomogram for 3-year T2DM risk in the training cohort (**a**) and validation cohort (**b**). *The blue line represents the net benefit when no participant was considered to exhibit T2DM, while the green line represents the net benefit when all participants were considered to suffer from T2DM. The area among the model curve, “treat none line” (blue line) and “treat all line” (green line), represents the clinical usefulness of the model. The farther the model curve is to the blue and green lines, the better clinical value the nomogram holds. *Using bootstrap resampling (times = 500)

In the model-development phase, according to the Akaike information criterion, we performed a backward step-down selection process by a threshold of *P*  <  0.05 to establish a parsimonious model (stepwise model) and formulated a nomogram in the training cohort (Fig. 2). The receiver operating characteristic (ROC) curves were plotted (Fig. 3), and the area under the ROC curve (AUC) was calculated (Table 4). To evaluate the discriminatory ability of the nomogram, we computed the AUC with a 95% CI by using 500 bootstrap resamplings. Sensitivity, specificity, positive predictive value (PPV), negative predictive value (NPV), positive likelihood ratio (PLR), and negative likelihood ratio (NLR) of the stepwise model are also presented in Table 4. Decision curve analysis [14] was performed to determine the clinical usefulness of the model: the proportion of the person who showed a true positive result subtracted by the proportion of the person who showed the false positive result, and then weighed the relative hazard of the false positive and false negative results to obtain a net benefit of making a decision (Fig. 4). We also established a full model and a multivariable fractional polynomial (MFP) model; nomograms and predictive accuracy are presented in Supplemental Appendix (Fig. S1 and S2; Table S1). In addition, we conducted ROC analyses to determine the optimal cutoff values of each risk factor; optimal cutoff values were defined as the points on the ROC curve where Youden’s index (sensitivity + specificity − 1) was the highest (Table 5). The ROC curves of each risk factor are presented in Supplemental Appendix (Fig. S3). The statistical analyses were 2-tailed, and *P* value < 0.05 was considered statistically significant. All the statistical analyses were performed with statistical packages R (http://www.R-project.org) and EmpowerStats (www.empowerstats.com, X&Y Solutions, Inc., Boston, MA).

**Table 4:** Prediction performance of the nomogram for estimating the 3-year risk of T2DM*

	Female		Male	
	Training cohort	Validation cohort	Training cohort	Validation cohort
AUC (95% CI)	0.863 (0.837, 0.888)	0.847 (0.801, 0.892)	0.751 (0.729, 0.774)	0.755 (0.717, 0.793)
Cutoff value	− 2.45	− 3.05	− 1.94	− 1.62
Sensitivity, %	82.63	90.24	73.81	63.33
Specificity, %	79.07	66.71	65.68	76.01
PPV, %	24.04	13.86	24.83	29.05
NPV, %	98.27	99.14	94.23	93.04
PLR	3.95	2.71	2.15	2.64
NLR	0.21	0.15	0.40	0.48

*AUC* area under curve, *PPV* positive predictive value, *NPV* negative predictive value, *PLR* positive likelihood ratio, *NLR* negative likelihood ratio

*Using bootstrap resampling (times = 500)

**Table 5:** Optimal cutoff values of risk factors for T2DM* (*N* = 5557)

	Female				Male			
Characteristic*	Cutoff value	AUC	Sensitivity (%)	Specificity (%)	Cutoff value	AUC	Sensitivity (%)	Specificity (%)
Age (years)	47.5	0.802	78.19	70.75	46.5	0.663	63.55	59.88
BMI (kg/m^2^)	22.9	0.681	65.99	63.15	23.70	0.635	75.60	42.79
FBG (mmol/L)	5.06	0.701	62.75	67.26	5.37	0.657	40.57	81.42
SBP (mmHg)	117.50	0.706	66.79	67.51	125.50	0.586	46.49	70.01
DBP (mmHg)	70.50	0.610	69.74	54.79	84.50	0.551	40.00	70.54
TC (mmol/L)	5.06	0.678	68.35	68.00	4.94	0.589	62.45	59.52
LDLc (mmol/L)	2.63	0.632	63.50	68.77	2.54	0.546	61.79	52.60
HDLc (mmol/L)	1.53	0.539	52.55	57.29	1.34	0.550	46.88	62.39
TG (mmol/L)	1.07	0.754	84.85	56.19	1.65	0.648	66.83	60.95

*T2DM* type 2 diabetes mellitus, *FBG* fasting blood-glucose, *PG2h* 2-h postprandial plasma glucose, *SBP* systolic blood pressure, *DBP* diastolic blood pressure, *TC* total cholesterol, *HDLc* high-density lipoprotein cholesterol, *LDLc* low-density lipoprotein cholesterol, *TG* triglycerides

*Using bootstrap resampling (times = 500)

## Results

### Baseline characteristics and risk factors of T2DM

After a 3-year follow up, 5557 and 1870 individuals were included in the final analysis as the training and validation cohorts, respectively. Approximately 10.71% and 11.02% of participants developed T2DM in the training and validation cohorts, respectively. The difference between the two sets was insignificant (Table 1). For the training cohort, Table 2 displays the baseline characteristics of participants grouped as those with or without incident T2DM. Significant differences were observed: patients with T2DM showed significantly high baseline age, BMI, FBG, blood pressure, TC, LDLc, and TG levels and low baseline HDLc levels, and were likely to be male. Univariate logistic regression analysis also reached similar conclusions.

In the multivariate logistic analyses, on the basis of the odds ratio (95% CI) and *P* value results, age, BMI, FBG, and TG were significantly associated with high risk of T2DM in both sexes. In addition, HDLc was observed to have a significant negative correlation with the risk of T2DM in males but not in females (Table 3).

### Development and validation of a T2DM-predicting nomogram

The nomogram of the stepwise model was drawn to provide a quantitative and convenient tool in predicting the risk of T2DM by using age, BMI, FBG, LDLc, HDLc, and TG in the training cohort (Fig. 2). To estimate an individual’s 3-year risk of T2DM, his/her value is located on each variable axis. A vertical line is drawn from that value to the top Points scale for determining how many points are assigned by that variable value. Then, the points from each variable value are summed. The sum is located at the Total Points scale and is vertically projected onto the bottom axis, thus obtaining a personalized 3-year risk of T2DM.

The prediction accuracy of the nomogram is presented in Table 4, and ROC curves are shown in Fig. 3. The resulting model was internally validated by 500 bootstrap resamplings. In the training cohort, the areas under the ROC curve (AUCs) of females and males were 0.863 (95% CI, 0.837–0.888) and 0.751 (95% CI, 0.729–0.774), respectively. The AUC value of females was higher than that of males. A similar result was observed in the subsequent validation cohort. The nomogram displayed AUCs of 0.847 (95% CI, 0.801–0.892) and 0.755 (95% CI, 0.717–0.794) for females and males, respectively. The optimal cutoff value of the nomogram was − 2.446 for females and − 1.942 for males in the training cohort and − 3.050 for females and − 1.619 for males in the validation cohort. In the training cohort, the sensitivity rates were 82.6% and 73.8%, and the specificity percentages were 79.1% and 65.7% for females and males, respectively. In the validation cohort, the sensitivity rates were 90.2% and 63.3%, and the specificity percentages were 66.7% and 76.0% for females and males, respectively. Notably, both the training and validation cohorts showed relatively high NPV. In summary, the nomogram demonstrated good predictive accuracy in estimating the risk of T2DM both in female and male subjects.

### Decision curves for the T2DM-predicting nomogram

Figure 4 illustrates the decision curves for the training and validation cohorts to predict the incidence of T2DM. The blue line represents the net benefit when no participant was considered to exhibit T2DM, while the green line represents the net benefit when all participants were considered to suffer from T2DM. The area among the model curve, “treat none line” (blue line) and “treat all line” (green line), represents the clinical usefulness of the model. The farther the model curve is to the blue and green lines, the better clinical value the nomogram holds. For example, in the training cohort, at the 10% risk cutoff, the net benefit was about 50% for males, which was equivalent to performing 50 further T2DM screenings (OGTT) per 100 men without the negative incidence of T2DM in the next 3 years.

### Optimal cutoff values of risk factors for T2DM prediction

The optimal cutoff values of each risk factor that was determined using the ROC analyses in both sexes are summarized in Table 5. The cutoff values of age, BMI, FBG, systolic blood pressure (SBP), diastolic blood pressure (DBP), TC, LDLc, HDLc, and TG were 47.5 and 46.5 years, 22.9 and 23.7 kg/m^2^, 5.1 and 5.4 mmol/L, 118 and 123 mmHg, 71 and 85 mmHg, 5.06 and 4.94 mmol/L, 2.63 and 2.54 mmol/L, 1.53 and 1.34 mmol/L, and 1.07 and 1.65 mmol/L for females and males, respectively, to predict the 3-year risk of T2DM optimally.

## Discussion

In this community-based cohort study, we developed a quantifiable and simple nomogram to predict the 3-year risk of T2DM in Central Chinese residents. After an internal validation, high degrees of predictive accuracy were found in both training and validation cohorts. Decision curve analysis also demonstrated the clinical value of this nomogram. We also estimated the optimal cutoff values of each risk factor for T2DM prediction. To the best of our knowledge, this study is the first to develop a nomogram by using continuous values instead of segmented values in estimating the T2DM risk in China. Moreover, the nomogram will also be of considerable practical value for its readily obtained parameters.

Different diabetes prediction models on the basis of demographic information and clinical measurements have been developed in European, North American [9, 10], and Asian populations [15, 16]. Given the genetic and environmental differences (i.e., economic level, diet, lifestyle, climate), risk factors for T2DM vary in terms of intensity or distribution across different populations, which suggested that a predictive model may not perform well in other ethnic groups [7, 17], even in individuals of the same ethnic group living in different cultural settings [16]. For the Chinese population, a limited number of T2DM risk prediction models was developed. In 2009, Chien et al. [8] constructed a simple point model for the prediction of diabetes incidence in Taiwan, with the estimated AUC value of 0.702 (95% CI, 0.676–0.727). In 2010, Ko et al. [12] developed a simple risk score to identify the young-to-middle-aged Chinese population at high risk for diabetes in Hong Kong, with the AUC value of 0.735 (95% CI, 0.705–0.765). In 2011, Chuang et al. [18] provided multiple diabetic prediction equations, which were derived from large-scale health check-up data that estimated the risk of diabetes in Taiwan. In 2014, Xu et al. [11] demonstrated that the Framingham diabetes score underestimated diabetes incidence in older Chinese populations and constructed a prediction model for this population in Guangzhou (AUC = 0.779, 95% CI, 0.756–0.801). In 2017, Chen et al. [19] developed a noninvasive T2DM risk score model for rural adults in Deqing.

All of the studies conducted in China mentioned above were carried out in Southern and Eastern China. Considering the large diversity of the Chinese population, T2DM prediction models that reflect regional characteristics will be needed in other parts of China. Our study filled this gap. This nomogram will be applicable to most individuals in Central China. In addition, all of the previously conducted T2DM risk prediction studies in China established T2DM risk scores with integer point or segment values, whereas our nomogram can provide more accurate and individualized risk predictions because of the use of continuous values, which is in line with the EPMA’s view that individualization should be a general societal trend in medicine and healthcare [3, 4].

In our study, good degrees of discrimination and prediction ability were found both in the training cohort (AUC = 0.863 for females, AUC = 0.751 for males) and validation cohort (AUC = 0.847 for females, AUC = 0.755 for males), which indicated a relatively good predictive capability to discriminate individuals who are at risk to develop T2DM from those who are not. The AUC values of females were larger than those of males in this study; this result was consistent with some previous similar studies [20, 21]. Decision curve analysis demonstrated that the nomogram can avoid the need to perform OGTT in individuals with a low risk of developing T2DM in 3 years, which relieved both burden and costs. In addition, the analysis of a model by using all risk factors (i.e., full model) showed that including SBP and DBP did not improve prediction. The MFP model showed slightly better accuracy than the stepwise model, but the complicated formula restricted its use. (Details are described in the Supplemental Appendix.) Therefore, the stepwise model is the simplest model under the premise of guaranteeing accuracy.

The parameters included in this nomogram for females and males were age, BMI, FBG, LDLc, HDLc, and TG, which were also included in other prediction models [8, 10]. Considerable research has proved that advanced age is a nonmodifiable risk factor for diabetes manifestation [3]. Impaired FBG is one of the diagnostic criteria for diabetes; studies have shown that FBG, hemoglobin A1c, and 2hPG all predict diabetes mellitus, yet test reliability is better for FBG and hemoglobin A1c than for 2hPG [22]. Moreover, compared with hemoglobin A1c, the feasibility and applicability of FBG testing in low-resource settings are more pronounced. Multiple studies have found that obesity [23], dyslipidemia [24], and T2DM conditions typically co-exist in an individual and share common pathological mechanisms [23, 25] (insulin resistance, metabolic disorders, inflammation and alteration of gut microbiota, etc.). Therefore, the application of these parameters for this model is well-founded.

Some known risk factors of T2DM, such as dietary habits and physical inactivity, were not included in this study because they are difficult to assess precisely. Similarly, smoking status was excluded because of the wide presence of passive smoking. Given the insufficiency in the health system of China in the last century, many people possessed undiagnosed diabetes. Therefore, many participants are unsure of the medical history of the previous generation of their family. Therefore, we did not collect data on family history. In addition, Chien et al. [8] demonstrated that lifestyle factors were the insignificant predictors of T2DM, and the parameter of family history cannot improve risk prediction. Chung et al. [15] and Poltavskiy et al. [26] also suggested that physical activity was unrelated to T2DM risk or does not significantly affect the prediction of undiagnosed prediabetes.

The current study did not collect information on 2hPG, insulin resistance, and genetic markers because these tests are expensive, time-consuming, and not routinely measured in clinical practice. Moreover, the addition of 2hPG and insulin resistance was found to not improve the risk prediction accuracy of a simple clinical model [9, 27]. Noble et al. [28] also suggested that sociodemographic and clinical data are much better predictors for the risk of diabetes than genetic markers.

Developing a nomogram for T2DM by using parameters that can be collected in general health care settings results in considerable clinical and social implications, especially for the residents in mainland China, where OGTT is not easily accessible. Our nomogram presents a quantitative approach to distinguish the high-risk groups of T2DM who must focus on their physical conditions and follow advanced intervention strategies (e.g., lifestyle interventions, appropriate drug interventions, and/or surgical interventions) [5, 6] to prevent or at least delay the onset of T2DM. Meanwhile, further T2DM screening (OGTT) is unnecessary for those with low risk of T2DM at present, which may increase the cost-effectiveness of T2DM screening. This simple risk assessment tool can be accepted by nonprofessional personnel and healthcare workers.

Besides, we used a relatively large community-based sample to estimate the optimal cutoff values of each risk factor to predict T2DM incidence, which may provide references in defining the best thresholds of age, BMI, FBG, and serum lipid parameters for the Chinese population.

The strength of our study was the fact that the nomogram was constructed from a large physical examination data of community-dwelling residents, which can provide good generalizability to the rest of the population. The limitation of this study was the fact that we did not collect information on waist circumference, which was included in some previous similar studies. However, the AUCs of our nomogram without waist circumference remained satisfactory. In addition, the studies of Framingham [9], along with the study of Chien [8] and Xu [11], all found that including waist circumference instead of BMI in the model cannot improve the prediction performance.

## Expert recommendations

A Special Session “PPPM in Diabetes Mellitus” of 2012 EPMA White Paper [4] suggested that behavioral modifications and promotion of adopting healthier dietary habits and lifestyle choices must be initiated early in life. An EPMA position paper 2016 [29] highlights the importance of an effective implementation of PPPM concepts to diabetes care, and emphasizes the need to address the all-encompassing complex approach for population screening; primary, secondary, and tertiary care benefiting nondiseased individuals; and predisposed subgroups. Our personalized T2DM prediction model rooting in the advanced healthcare concept of PPPM gives very promising results. For follow-up developments, we will further optimize the model in conjunction with additional tools such as circulation biomarkers (pancreatic polypeptide-specific antigens [30], etc.) and suboptimal health status [31] for follow-up developments.

## Conclusion

In conclusion, our nomogram can be used as a simple, plausible, affordable, and widely implementable tool to predict the 3-year risk of T2DM for the residents in Central China. Considering the large number of patients with diabetes and high medical cost of diabetes care in China, the successful identification of at-risk individuals and intervention at the early stage can alleviate the burden of this disease and provide advanced strategies from a PPPM perspective.

## Electronic supplementary material

ESM 1 (DOCX 688 kb)

## Data Availability

The datasets generated during the current study are available from the corresponding author on reasonable request.

## Abbreviations

T2DM: type 2 diabetes mellitus
PPPM: predictive, preventive, and personalized medicine
EPMA: European Association for Predictive, Preventive and Personalised Medicine BMI body mass index
TC: total cholesterol
LDLc: low-density lipoprotein cholesterol
HDLc: high-density lipoprotein cholesterol
TG: triglycerides
OGTT: oral glucose tolerance test
FBG: fasting blood glucose
2hPG: 2-h postprandial plasma glucose
ANOVA: analysis of variance
ROC: receiver operating characteristic
AUC: area under the ROC curve
CI: confidence interval
PPV: positive predictive value
NPV: negative predictive value
PLR: positive likelihood ratio
NLR: negative likelihood ratio
