# Supplementary material for: Nomogram prediction for the 3-year risk of type 2 diabetes in healthy mainland China residents
Source: EPMA J. 2019 Aug 6;10(3):227–37. doi: 10.1007/s13167-019-00181-2 (PMC6695459; doi:10.1007/s13167-019-00181-2)
Supplement: Supplementary file 1 — (DOCX 688 kb) [file 13167_2019_181_MOESM1_ESM.docx]

**
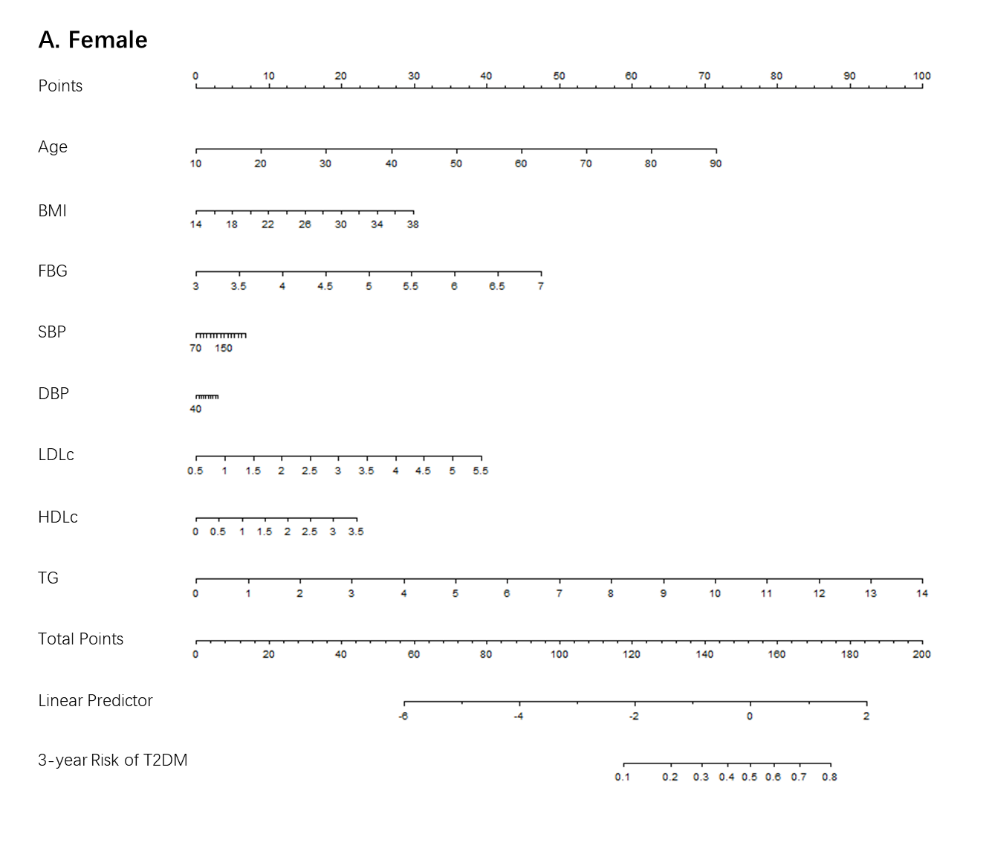
**

**
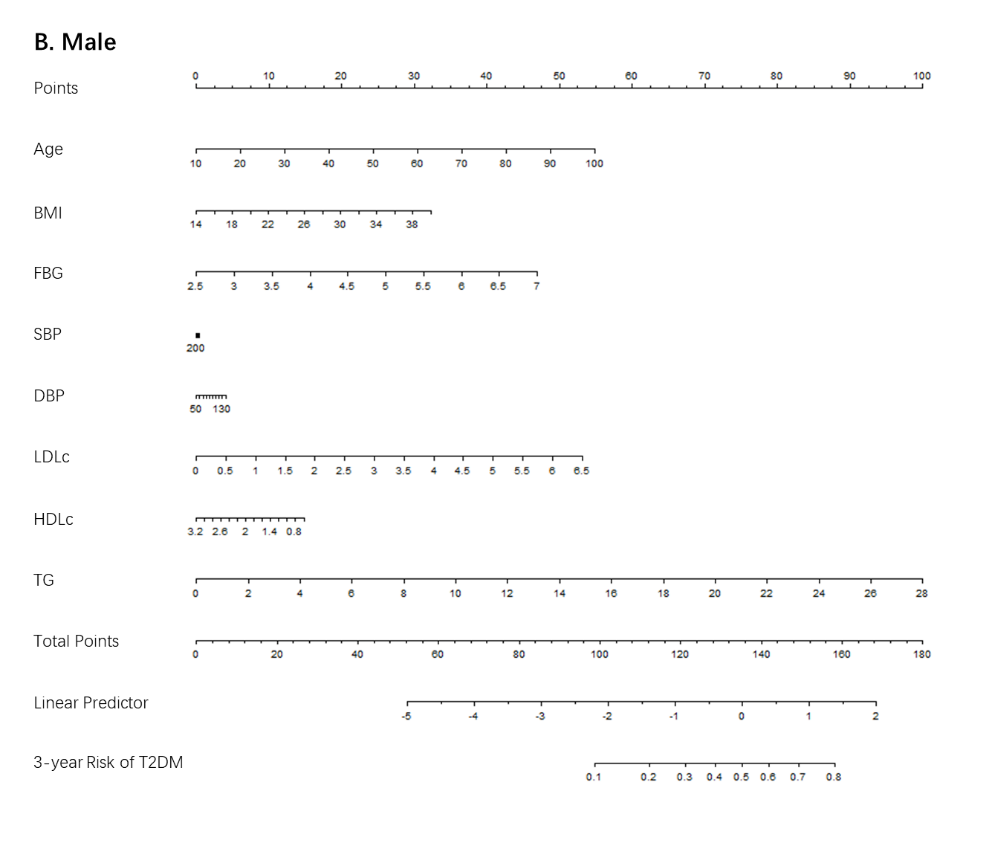
**

**Fig. S1: Full model Nomogram to predict the 3-year risk of T2DM for female and male. ***

Instructions: to estimate a patient’s 3-year risk of T2DM, locate the individual’s value on each variable axis. Draw a vertical line from that value to the top Points scale for determining how many points are assigned by that variable value. Then, the points from each variable value are summed. Locate the sum on the Total Points scale and vertically project it onto the bottom axis, thus obtaining a personalized 3-year risk of T2DM.

*****Using Bootstrap resampling (times = 500)

**
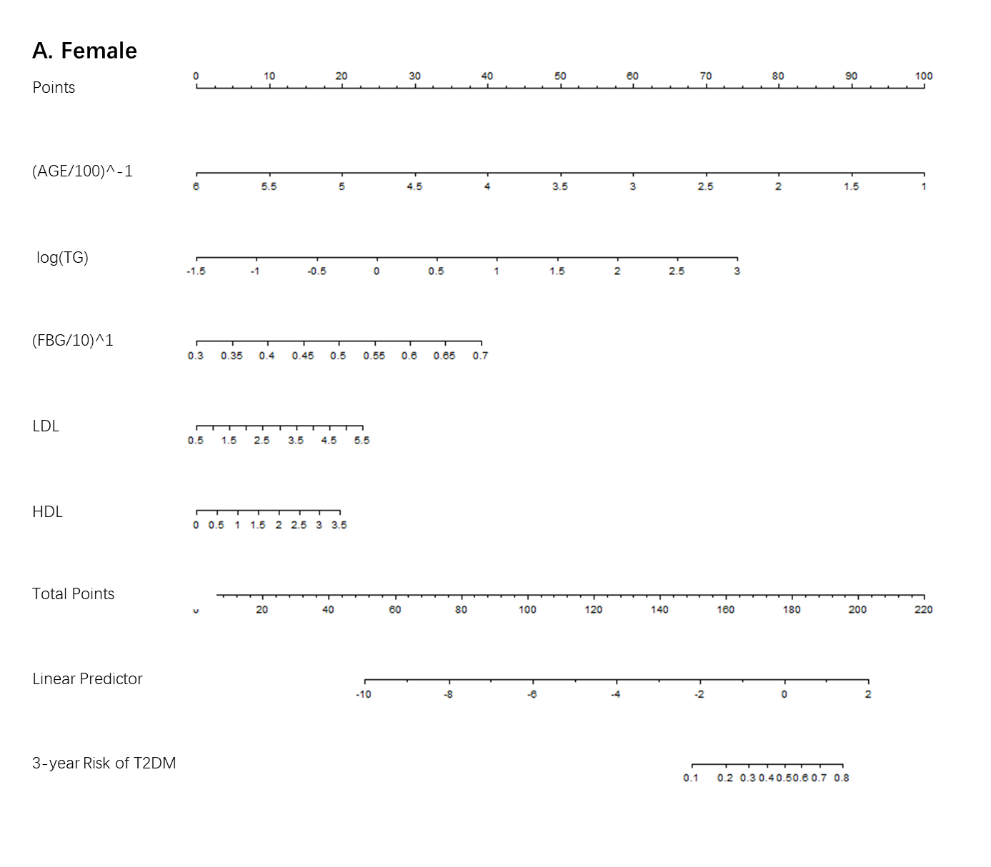
**

**
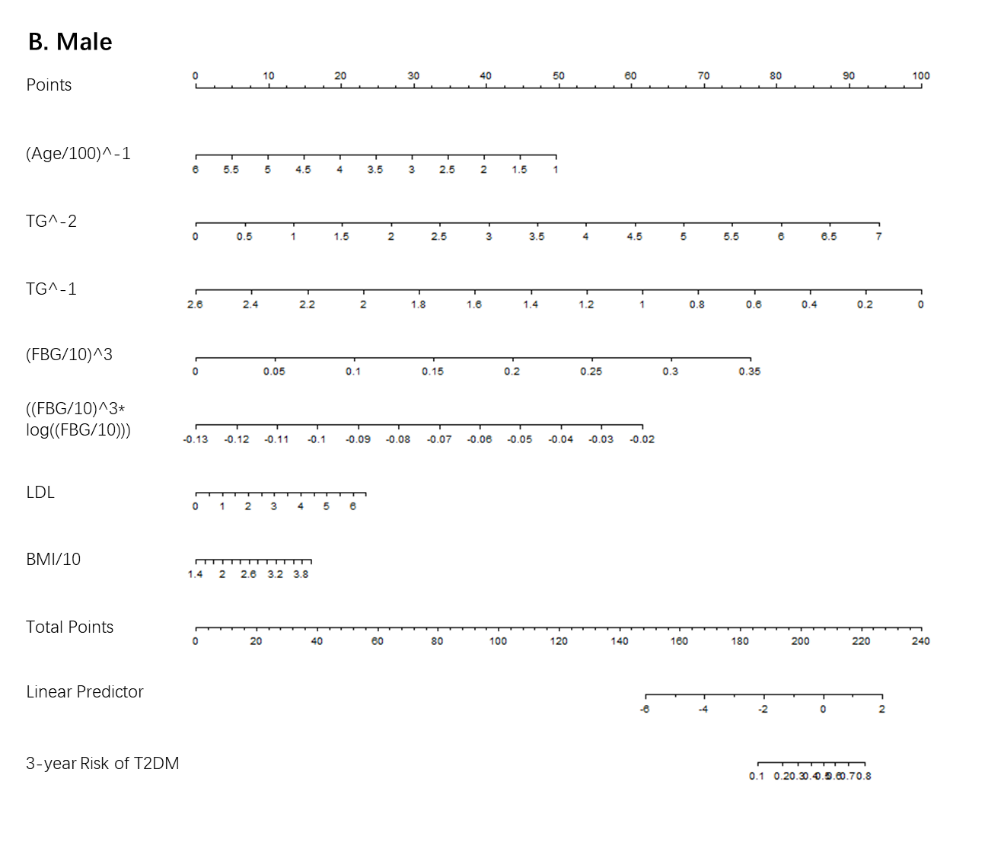
**

**Fig. S2: MFP model Nomogram to predict the 3-year risk of T2DM for female and male. ***

Instructions: to estimate a patient’s 3-year risk of T2DM, locate the individual’s value on each variable axis. Draw a vertical line from that value to the top Points scale for determining how many points are assigned by that variable value. Then, the points from each variable value are summed. Locate the sum on the Total Points scale and vertically project it onto the bottom axis, thus obtaining a personalized 3-year risk of T2DM. *****Using Bootstrap resampling (times = 500)

**Table S1. Prediction Performance of Three Prediction Models for Estimating the 3-year Risk of T2DM.***

|  | **MFP model** | | **Stepwise model** | | **Full model** | |
| --- | --- | --- | --- | --- | --- | --- |
|  | **Training cohort** | **Validation cohort** | **Training cohort** | **Validation cohort** | **Training cohort** | **Validation cohort** |
| **Female** | | | | | | |
| **AUC**  **(95% CI)** | 0.8645  (0.8396, 0.8893) | 0.8611  (0.8175, 0.9047) | 0.8627  (0.8371, 0.8884) | 0.8465  (0.8013, 0.8916) | 0.8635  (0.8384, 0.8886) | 0.8424  (0.7963, 0.8885) |
| **Cut-off value** | -2.4229 | -2.6986 | -2.4460 | -3.0495 | -2.6548 | -3.1987 |
| **Sensitivity, %** | 83.83 | 90.24 | 82.63 | 90.24 | 85.63 | 92.68 |
| **Specificity, %** | 76.76 | 71.78 | 79.07 | 66.71 | 74.75 | 63.82 |
| **PPV, %** | 22.44 | 15.95 | 24.04 | 13.86 | 21.38 | 13.19 |
| **NPV, %** | 98.34 | 99.20 | 98.27 | 99.14 | 98.48 | 99.32 |
| **Male** | | | | | | |
| **AUC**  **(95% CI)** | 0.7787  (0.7574, 0.8000) | 0.7698  (0.7341，0.8056) | 0.7513  (0.7285, 0.7742) | 0.7553  (0.7169, 0.7938) | 0.7520  (0.7292, 0.7747) | 0.7556  (0.7173, 0.7938) |
| **Cut-off value** | -1.9417 | -2.0836 | -1.9416 | -1.6191 | -1.9825 | -1.6159 |
| **Sensitivity, %** | 78.56 | 82.67 | 73.81 | 63.33 | 75.17 | 63.33 |
| **Specificity, %** | 65.65 | 60.60 | 65.68 | 76.01 | 64.26 | 76.42 |
| **PPV, %** | 25.99 | 24.55 | 24.83 | 29.05 | 24.41 | 29.41 |
| **NPV, %** | 95.22 | 95.75 | 94.23 | 93.04 | 94.40 | 93.07 |

**For female:**

**MFP model:** -5.08623 -1.57802*((Age/100)^-1) +1.30337*log(TG) +7.71208*((FBG/10)^1) +0.35948*(LDLc^1) +0.44450*(HDLc^1)

**Stepwise model:** -14.46495 +0.05684*Age +0.09344*BMI +0.75239*FBG +0.52808*LDLc +0.61667*HDLc +0.49127*TG

**Full model:** -14.16760 +0.05667*Age +0.07576*BMI +0.75943*FBG +0.00312*SBP +0.00229*DBP +0.50394*LDLc +0.39551*HDLc +0.46299*TG

**For male:**

**MFP model:** 1.64982 -1.01199*((Age/100)^-1) +1.37180*(TG^-2) -3.92611*(TG^-1) +22.27016*((FBG/10)^3) +57.02244*((FBG/10)^3 * log((FBG/10))) +0.36784*(LDLc^1) +0.62280*((BMI/10)^1)

**Stepwise model:** -9.85474 +0.03766*Age +0.07676*BMI +0.62260*FBG +0.50349*LDLc -0.46460*HDLc +0.20988*TG

**Full model:** -10.14300 +0.03721*Age +0.07595*BMI +0.61570*FBG -0.00030*SBP +0.00293*DBP +0.49564*LDLc -0.35304*HDLc +0.22014*TG

AUC: area under curve.

*****Using Bootstrap resampling (times = 500)


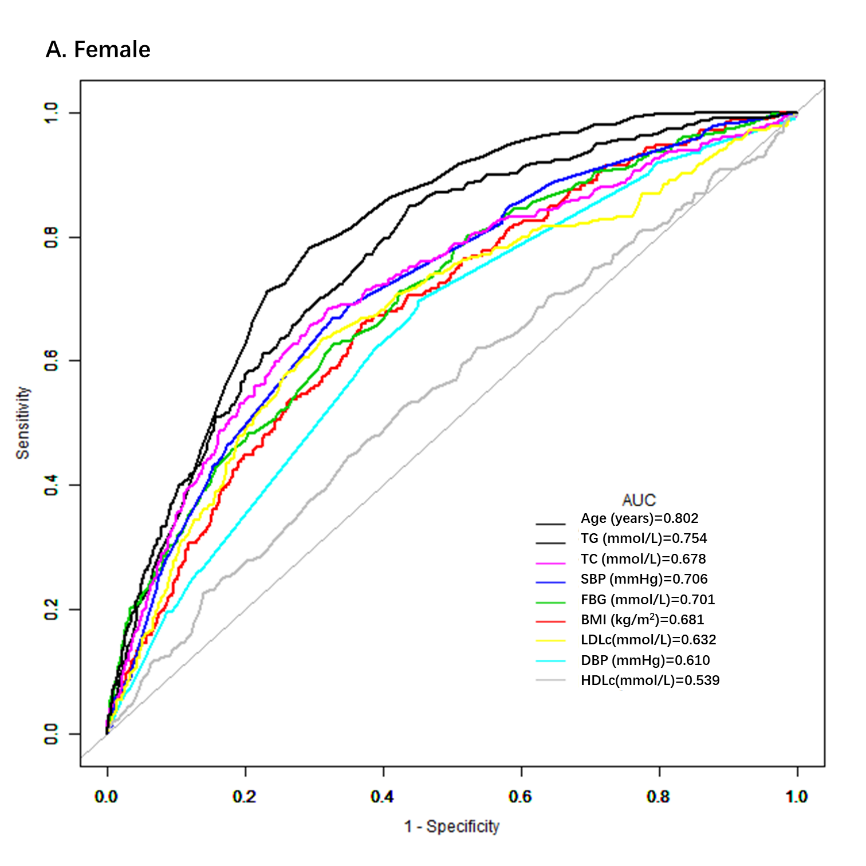

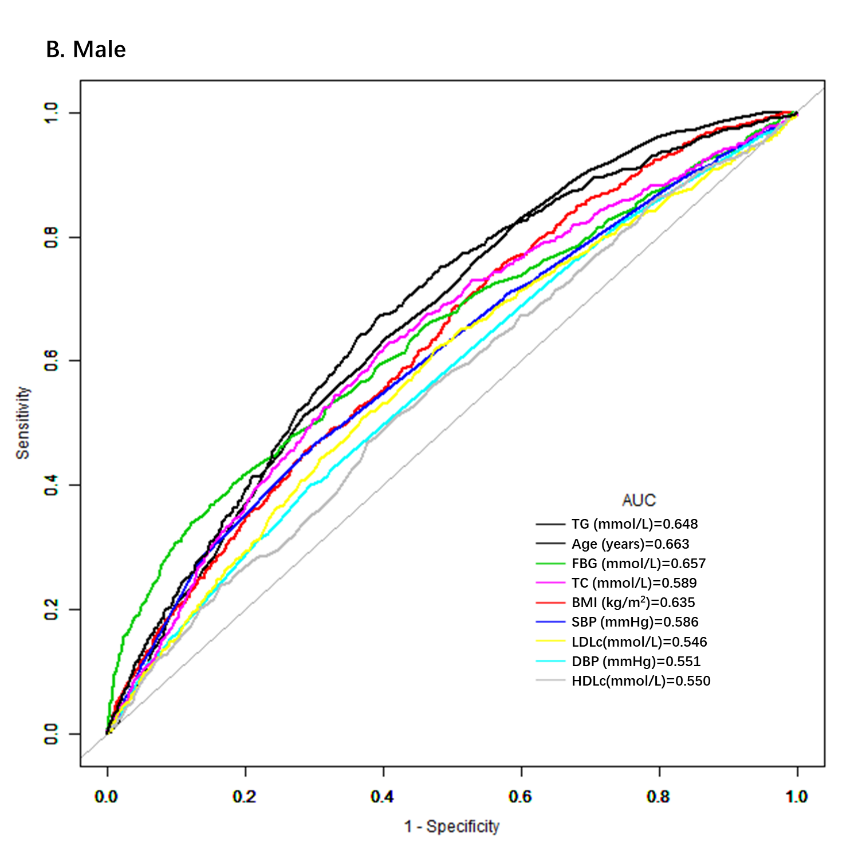


**Fig. S3. ROC curve of each risk factor for 3-year T2DM risk.**
